# Pathogens in PICU before and during the SARS-CoV-2 pandemic in China: a multicenter retrospective study

**DOI:** 10.1186/s12879-023-08687-x

**Published:** 2023-10-20

**Authors:** Jingwen Ni, Zhe Zhao, Chun Wang, Youpeng Jin, Yi Wang, Zhenhua Liang, Shujun Li, Jie Chen, Yanqiang Du, Yipei Li, Hanwu Huang, Yuxiong Guo, Yujie Zhong, Zhichun Feng, Kenan Fang, Xiaoyang Hong

**Affiliations:** 1 Pediatric Intensive Care Unit, Luoyang Maternal and Child Health Hospital, Luoyang, China; 2 Pediatric Intensive Care Unit, Faculty of Pediatrics, The Seventh Medical Center of Chinese PLA General Hospital, Beijing, China; 3 Pediatric Intensive Care Unit, Department of Pediatrics, Guangdong Provincial People’s Hospital, Guangdong Academy of Medical Sciences, Guangzhou, China; 4 Pediatric Intensive Care Unit, Shandong Provincial Hospital Affiliated to Shandong First Medical University, Jinan, China; 5 Pediatric Intensive Care Unit, Shandong Provincial Hospital, Cheeloo College of Medicine, Shandong University, Jinan, China; 6 Pediatric Intensive Care Unit, National Children’s Regional Medical Center (Northwest), Xi’an, China; 7 Children’s Hospital of Xi’an Jiaotong University, Xi’an, China; 8 Xi’an Children’s Hospital, Xi’an, China; 9 Pediatric Intensive Care Unit, The People’s Hospital of Guangxi Zhuang Autonomous Region, Nanning, Guangxi China; 10 Pediatric Intensive Care Unit, The First Affiliated Hospital of Xinxiang Medical University, Xinxiang, China; 11 Pediatric Intensive Care Unit, Shandong Provincial Hospital, Jinan, China; 12 Shandong University, Jinan, China; 13 The second school of Clinical Medicine, Southern Medical University, Guangdong, China; 14 Laboratory, Luoyang Maternal and Child Health Hospital, Luoyang, China

**Keywords:** COVID-19, Pediatric intensive care units, Bacterial Infection

## Abstract

**Background:**

Nonpharmacological interventions for COVID-19 could reduce the incidence of children hospitalized in pediatric intensive care units (PICU) and the incidence of children with bacterial infections. This study aimed to evaluate changes in the bacterial profile of children in PICU before and during the COVID-19 pandemics.

**Methods:**

This is a retrospective study, involving clinical data of children with positive bacterial cultures admitted to the PICU respectively in 2019 and 2021.

**Results:**

In total 652 children were included in this study. The total number of hospitalized patients and the incidence of bacteria-positive children in 2021 were lower than those in 2019. There were no significant differences in the ratio of Gram-positive bacterial infection, Gram-negative bacteria infection or fungi infection between the two years. The rate of *Streptococcus pneumoniae* in 2021 was higher than that in 2019(p = 0.127). The incidence of *Haemophilus influenzae* in hospitalized patients decreased with a downward trend(p = 0.002). The distribution of previous underlying diseases in children admitted to PICU with different outcomes of bacterial infection between the two years were homogeneous (p > 0.05).

**Conclusion:**

After the implementation of COVID-19 isolation, prevention and control measures, the number of hospitalizations and bacterial infections in PICU decreased, which may be due to changes in population’s behavior patterns. Meanwhile, the incidence of *Haemophilus influenzae* in hospitalized patients decreased with a downward trend.

## Introduction

COVID-19 caused by SARS-CoV-2 infection broke out in Wuhan, China at the end of 2019. Protective measures such as large-scale control of the movement of population, large-scale disinfection, suspension of school, work and maximum use of masks to cut off the transmission of the coronavirus to the greatest extent were implemented. After applying a series of prevention and control measures, though the spread of the coronavirus has been controlled, it is still prevalent within a certain range.

A United States multi-center study showed that between March 2020 and April 2020, the incidence of acute respiratory illnesses and acute respiratory infection involving respiratory syncytial virus and influenza in children in seven cities continued to decrease. This result may be attributed to timely and sustained isolation measures, which have effectively cut off transmission routes [1]. Children with severe infection admitted to pediatric intensive care unit (PICU) mostly suffer mixed infections of both virus and bacteria. Notably, bacterial infection has been considered as an important complication of influenza pandemic, among which *Streptococcus pneumoniae* infection is the most common [2].

Moreover, studies have demonstrated that influenza, as a risk factor for bacterial infection, can often lead to secondary bacterial infection of *Streptococcus pneumoniae*, *Staphylococcus aureus* or *Haemophilus influenzae* [3]. In the United States, the number of people vaccinated with pneumococcal vaccine and influenza vaccine decreased significantly during the COVID-19 pandemic [4]. However, taking pneumococcal vaccine as an example, vaccination is an important way to prevent its infection [4, 5]. Although the whole world is struggling to cope with the consequences of the global pandemic associated with severe pneumonia caused by SARS-CoV-2, the existing evidences suggest that bacterial infection is a key factor contributing to severe infection in children. This study aimed to evaluate whether the bacterial pathogens in PICU inpatients in Mainland China changed during the Covid-19 epidemic.

## Methods

This is a retrospective study. The data of six representative medical centers in different regions of China were collected, namely: Luoyang Maternal and Child Health Hospital, Henan Province (Central China), The Seventh Medical Center of Chinese PLA General Hospital (North China), Guangdong Provincial People’s Hospital, and The People’s Hospital of Guangxi Zhuang Autonomous Region (South China), Shandong Provincial Hospital (East China), Xi’an Children’s Hospital (West China). Study subjects: the data of children within 48 h of PICU admission from 6 medical centers were evaluated. Inclusion criteria: (1) The patients involved in our research ranged from 29 days to 18 years; (2) Research period in: 2019 and 2021; (3) Children with positive bacterial culture results in the first 48 h of admission; (4) All the children who were admitted to the PICU, including those who were admitted directly to the PICU, and later needed to be admitted to the PICU. The specimens included sputum, tracheal aspirate, nasopharyngeal aspirate, bronchoalveolar lavage fluid, urine, cerebrospinal fluid and blood. Exclusion criteria: (1) Nosocomial infection (The infection occurred within 48 h after into the PICU; The infection directly related to the last hospitalization; A new infection in other parts on the basis of the original infection appeared); (2) The patient was admitted to the PICU after being in the ward for more than 48 h and there was growth in the culture after 48 h of hospitalization; (3) Repeated specimens from the same patient. All laboratory results came from the clinical laboratory management system, data were recorded by a physician on the preprinted case report forms and then collected by another physician with an electronical form. The data directly extracted from the electronical system was validated by two physicians back-to-back. The study was accorded to the approval of the Ethics Committee of Luoyang Maternal and Child Health Hospital, informed consent is not required(KY2022021401.0)(Luoyang; Henan, China). This study was registered at http://www.chictr.org.cn/index.aspx (ChiCTR2200057182).

### Statistical analysis

The descriptive variants of all the patient admitted to the PICUs was shown as frequency (percentage) for categorical variables or median (interquartile range) for continuous variables. The statistical analysis was performed with SPSS 24.0 (SPSS Inc., Chicago, USA). The Wilcoxon signed-rank test were used to compare the age, body weight, hospital duration and hospital cost between the two years. The categorical variables including the gender, whether the patients received mechanical ventilation, prognosis and the others were compared by the Cochran-Mantel-Haenszel (CMH) Chi-Square Test. The chi-square test was used to compare the rate of the *Streptococcus pneumoniae* in Gram positive bacteria and the *Haemophilus influenzae* in Gram negative bacteria.

## Results

**Fig. 1:**
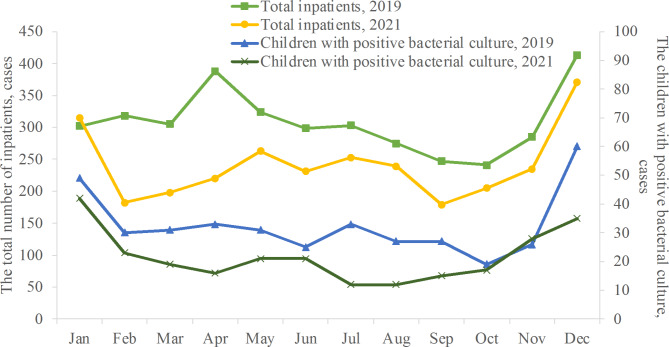
Monthly distribution of total inpatients and infections in 2019 and 2021

In 2019 and 2021, 3700 and 2891 patients were admitted to PICU, including 391 in 2019 and 261 children with positive bacterial culture in 2021. The total number of inpatients and the number of children with positive bacterial culture in 2019 were higher than those in 2021, respectively. A horizontal comparison of the total number of inpatients and the number of children with positive bacterial culture in the same period by month showed that the total number of inpatients in January 2019 was slightly lower than that in 2021, and the monthly data from February to December 2019 were higher than those in 2021 (See Fig. 1).

The baseline characteristics of the children with positive bacterial culture in 2019 and 2021 in the 6 medical centers were analyzed without statistically significant differences between the two years: mean age (7 months vs. 8 months, p = 0.119), mean body weight (7.8 kg vs. 8.0 kg, p = 0.094), gender (male 52.7%, female 47.3% vs. male 48.3%, female 51.7%, p = 0.817), the rate of mechanical ventilation (52.7% vs. 48.3%, p = 0.306), length of hospital stay (12d vs. 11d, p = 0.709), hospitalization cost (30.4 thousand yuan vs. 26.4 thousand yuan, p = 0.727), mortality (14.8% vs19.2%, p = 0.178) (see Table 1).

**Table 1:** Clinical characteristics of the children with positive bacterial culture admitted to PICU in 2019 and 2021

	2019(n = 391)	2021(n = 261)	statistics	*P value*
**age**^**a**^, **month**	7.0 (2.8, 22.5)	8.0 (3.1, 36.0)	47354.5	0.119
**weight**^**a**^, **kg**	7.8 (5.0, 11.4)	8.0 (5.5, 14.0)	46,956	0.094
**sex** ^**b**^			0.054	0.817
male	237 (60.6)	155 (59.4)		
female	154 (39.4)	106 (40.6)		
**Mechanical ventilation** ^**b**^			1.048	0.306
yes	206 (52.7)	126 (48.3)		
no	185 (47.3)	135 (51.7)		
**Hospital stay**^**a**^, **day**	12.0 (7.0, 18.0)	11.0 (7.0, 21.0)	50146.5	0.709
**Hospital cost** ^**a**,^ **thousand RMB**	30.4 (12.4, 63.8)	26.4 (12.4, 69.8)	51,126	0.727
**outcome** ^**b**^			1.816	0.178
discharge	333 (85.2)	211 (80.8)		
death	58 (14.8)	50 (19.2)		

Note: a, continuous variable showed skewness distribution, which was expressed as median (Q1, Q3). Rank-sum test was used to compare the differences between the two years. b, categorical variable were expressed as n (%), and chi-square test was used to compare the differences between two years

Among the children with positive bacterial culture admitted to the PICUs in 2019 and 2021, the main source of positive results was respiratory tract. The distribution of infection sites showed no statistically significance between the two years (p = 0.467). In our cohort, infection sites include respiratory tract (78.3% vs. 82.8%), blood (11.5% vs. 10.3%), urinary system (2.3% vs. 1.9%), central nervous system (3.1% vs. 1.1%) and lung (4.9% vs. 3.8%) (see Table 2).

**Table 2:** Distribution of infection sites in children admitted to PICU in 2019 and 2021, n (%)

Infection sites	2019(n = 391)	2021(n = 261)	*χ* ^2^	*P*
**respiratory tract**	306 (78.3)	216 (82.8)	3.575	0.467
**blood**	45 (11.5)	27 (10.3)		
**urinary system**	9 (2.3)	5 (1.9)		
**central nervous system**	12 (3.1)	3 (1.1)		
**Lung**	19 (4.9)	10 (3.8)		

The rate of Gram-positive bacterial infection (40.4% vs. 42.1%, p = 0.659), Gram-negative bacterial infection (49.6% vs. 52.1%, p = 0.533), and fungi infection (10.0% vs. 5.7%, p = 0.055) in 2019 and 2021 showed no significant differences. The gram-positive bacteria included: *Streptococcus pneumoniae, Staphylococcus aureus*, *Enterococcus faecalis*, et al. The gram-negative bacteria included: *Haemophilus influenzae, Escherichia coli*, *Klebsiella pneumoniae*, et al. The fungi included as follows: *Candida*, *Yeast*, *Aspergillus*, *Sporoplasma*. The two-year comparison found that the rate of *Streptococcus pneumoniae* in Gram-positive bacteria in 2021 was higher than that in 2019, but without significant differences (23.4% vs. 31.8%, p = 0.127). Notably, among Gram-negative bacteria the rate of *Haemophilus influenzae* in 2021 was significantly lower than that in 2019 (14.9% vs. 4.4%, p = 0.002) (see Table 3).

**Table 3:** Changes in *Streptococcus pneumoniae* and *Haemophilus influenzae* in 2019 and 2021

Pathogens	2019(n = 391)	2021(n = 261)	*P* ^*a*^	*P* ^*b*^
**Gram positive**	**158 (40.4)**	**110 (42.1)**	**0.659**	**0.127**
*Streptococcus pneumoniae*	37 (23.4)	35 (31.8)		
**Gram negative**	**194 (49.6)**	**136 (52.1)**	**0.533**	**0.002**
*Haemophilus influenzae*	29 (14.9)	6 (4.4)		
**Fungus**	**39 (10.0)**	**15 (5.7)**	**0.055**	

Stratified analysis of clinical outcomes and former medical history of children with positive bacterial culture identified no significant differences in the rate of children discharged from hospital with former medical history between 2019 and 2021 (29.4% vs. 32.7%) (p = 0.420). The mortality in children with former medical history in 2021 was higher than that in 2019, though without statistically significant differences (44.0% vs. 37.9%, p = 0.522). The distribution of former medical history in children admitted to PICU with different pathogenic infections and clinical outcomes were homogenous (p > 0.05) (see Table 4).

**Table 4:** Stratified analysis of clinical outcomes and former medical history of children infected and admitted to PICU in 2019 and 2021

Outcome	Former medical history	2019	2021	*P*
**Discharge**	no	235 (70.6)	142 (67.3)	0.420
	yes	98 (29.4)	69 (32.7)	
**Death**	no	36 (62.1)	28 (56.0)	0.522
	yes	22 (37.9)	22 (44.0)	
**In Total**	no	271 (69.3)	170 (65.1)	0.264
	yes	120 (30.7)	91 (34.9)	

## Discussion

Children suspicious of severe bacterial infection in PICU are a frequent challenge for pediatricians, which if not handle with timely appropriate treatment would result in poor prognosis [6]. Bacterial infections in PICU include community acquired infection (CAI) and hospital acquired infection (HAI). This study collected data from patients with community-acquired bacterial infections admitted to PICUs in 6 representative medical centers from different regions of mainland China in 2019 and 2021.

This is a retrospective study based on infection distribution in the PICU of six medical centers and it revealed a decrease in the total number of children’s hospitalizations in 2021 compared to 2019. Studies have reported that quarantine prevention and control measures reduce COVID-19 cases. The timing of prevention and control strategies is strongly associated with the trending decline in the epidemic growth rate of COVID-19 cases [7–9]. While quarantine measures are implemented to contain the spread of COVID-19, the incidence of acute respiratory illnesses, respiratory syncytial virus and influenza acute respiratory illnesses in children also decreased [1]. The medical centers involved in this study were under the epidemic prevention and control measures in 2020. Thus, after 2020, the behavioral patterns of the people in 2021 have changed greatly from those in 2019. These conclusions of this study are consistent with previous reports that the number of hospitalizations reduced in 2021 as compared to 2019 might also be attributable to nationwide prevention and control measures, including control of the movement of people, mass disinfection, suspension of classes, work, and maximum use of masks and other protective measures. It should also be taken into consideration that the epidemic prevention and control measures have caused more patients to choose local hospitals for treatment, rather than being referred to higher-level hospitals across regions, which also explains the reduction in the number of hospitalizations.

In the present study, the number of bacterial infections in 2021 was lower than that in 2019. Of note, bacterial infection is an important complication of influenza pandemic [2]. Treatment of viral infection is anticipated to prevent bacterial superinfections, such as oral oseltamivir to anti influenza virus (type A and type B). Although the number of bacterial infections decreased in 2021 as compared with 2019, there was no difference in the distribution of infection sites, and the respiratory system was still the main infection site for bacterial infections in the PICU.

Previous studies have shown that *Haemophilus influenzae* and *Streptococcus pneumoniae* are the main pathogens in PICU [10]. Our study found a decrease in the rate of *Haemophilus influenzae* infections. *Haemophilus influenzae*, as an opportunistic pathogen, can cause a variety of clinical symptoms including otitis media, epiglottitis, sinusitis, and pneumonia, especially in children, the elderly, and immunocompromised patients. *Haemophilus influenzae* is mainly transmitted through respiratory droplets from carriers [11]. This result is consistent with the conclusions of previous studies [12, 13]. The various prevention and control measures during the epidemic could possibly cut off the pathogenic infection mainly dependent on the transmission of respiratory droplets [1]. However, a limitation of this study was that we failed to isolate the serotype of *Haemophilus influenzae*. In addition, unlike *Haemophilus influenzae*, our study found that the rate of *Streptococcus pneumoniae* infection in 2021 was higher than that in 2019. Previous studies have shown that for severe community acquired pneumonia patients in intensive care unit, influenza is a risk factor for bacterial infection and *Streptococcus pneumoniae* is the most common bacterium among the major complications of influenza pandemic [2]. Therefore, theoretically, *Streptococcus pneumoniae* infection secondary to virus infection should be reduced with declined rate of influenza, which is contrary to the results of this study. We speculate that the increase in *Streptococcus pneumoniae* infection is possibly relative to the reduced mass movement during the epidemic prevention and control measures, and the decreased vaccination of *Streptococcus pneumoniae* [3, 4]. In addition, CDC suggested that patients with positive diagnosis of covid-19 should delay routine immunization during the covid-19 pandemic [14]. However, due to the small sample size, no statistical difference between the two years was found. Therefore, a large sample size and long-term observation is necessary for evaluating the changes in *Streptococcus pneumoniae* infection over the years.

Moreover, we found that the mortality of children with former medical history was 31.9% in 2019 and 44.0% in 2021. Although the rate of hospitalization and bacterial infections in 2021 decreased, the mortality in children with former medical history increased, suggesting that epidemic prevention and control measures may be of some significance for the prevention of infectious diseases in healthy children, but children with previous medical history will need more protection and attention. However, due to the small sample size, there was no statistical difference between the two years, and further research and confirmation are needed for verification.

## Limitations

The study has several limitations. Firstly, this study of 3 years span cannot rule out the impact of improved technical improvements and detection rates during these three years. Secondly, this is a multi-center study with diversified distribution of cases provided by different research centers using differed detection methods, and the climate differences between regions may also affect pathogenic microorganisms, the impact of which on the present study have not been explored. Thirdly, disease spectrum and medical levels among medical centers are different, which may have an impact on mortality.

## Conclusions

After the implementation of COVID-19 isolation prevention and control measures, we noticed that with changed behavioral patterns in population, the number of PICU admissions and bacterial infections decreased, confirming that isolation and control measures can reduce admissions and bacterial infections in PICU. Meanwhile, the rate of *Haemophilus influenzae* infection in hospitalized patients demonstrated a downward trend. The rate of *Streptococcus pneumoniae* increased but without statistically significant differences due to the limited sample size, which requires further investigation.

## Data Availability

The datasets used and/or analyzed during the current study are available from the corresponding author on reasonable request.

## Abbreviations

SARS-CoV-2: Severe acute respiratory syndrome
COVID-19: Coronavirus 2, the cause of coronavirus disease 2019
PICU: Pediatric intensive care unit
CAI: Community acquired infection
HAI: Hospital acquired infection
